# The Texas Community-Engagement Research Alliance Against COVID-19 in Disproportionately Affected Communities (TX CEAL) Consortium

**DOI:** 10.1017/cts.2022.395

**Published:** 2022-04-25

**Authors:** Rebecca A. Seguin-Fowler, Chris Amos, Bettina M. Beech, Robert L. Ferrer, Lorna McNeill, Jasmine J. Opusunju, Emily Spence, Erika L. Thompson, Luis R. Torres-Hostos, Jamboor K. Vishwanatha

**Affiliations:** 1 Institute for Advancing Health through Agriculture, Texas A&M AgriLife, College Station, TX 77845, USA; 2 Baylor College of Medicine, Houston, TX 77030, USA; 3 Department of Health Systems and Population Health Sciences, University of Houston, Houston, TX 77004, USA; 4 University of Texas Health Science Center at San Antonio, Long School of Medicine, San Antonio, TX 78229, USA; 5 Department of Health Disparities Research, Division of Cancer Prevention and Population Sciences, MD Anderson Cancer Center, Houston, TX 77079, USA; 6 CAN DO Houston, Houston, TX 77012, USA; 7 The University of North Texas Health Science Center at Fort Worth, Fort Worth, TX 76107, USA; 8 Department of Biostatistics and Epidemiology, School of Public Health, The University of North Texas Health Science Center at Fort Worth, Fort Worth, TX 76107, USA; 9 School of Social Work, The University of Texas Rio Grande Valley, Edinburg, TX 78539, USA; 10 Graduate School of Biomedical Sciences, The University of North Texas Health Science Center at Fort Worth, Fort Worth, TX 76107, USA

**Keywords:** Coronavirus disease 2019 (COVID-19), vaccines, clinical trial, community engagement, prevention and treatment efforts, testing, barriers, awareness, education

## Abstract

The coronavirus disease 2019 (COVID-19) pandemic requires urgent implementation of effective community-engaged strategies to enhance education, awareness, and inclusion of underserved communities in prevention, mitigation, and treatment efforts. The Texas Community-Engagement Alliance Consortium was established with support from the United States’ National Institutes of Health (NIH) to conduct community-engaged projects in selected geographic locations with a high proportion of medically underserved minority groups with a disproportionate burden of COVID-19 disease and hospitalizations. The purpose of this paper is to describe the development of the Consortium. The Consortium organized seven projects with focused activities to address COVID-19 clinical and vaccine trials in highly affected counties, as well as critical statewide efforts. Five Texas counties (Bexar, Dallas, Harris, Hidalgo, and Tarrant) were chosen by NIH because of high concentrations of underserved minority communities, existing community infrastructure, ongoing efforts against COVID-19, and disproportionate burden of COVID-19. Policies and practices can contribute to disparities in COVID-19 risk, morbidity, and mortality. Community engagement is an essential element for effective public health strategies in medically underserved minority areas. Working with partners, the Consortium will use community engagement strategies to address COVID-19 disparities.

## Introduction

In response to the rapid acceleration of the coronavirus disease 19 (COVID-19) pandemic, the National Institutes of Health (NIH) initiated a nationwide research opportunity called ***C*** ommunity-***E*** ngagement ***Al*** liance (CEAL) against COVID-19 in disproportionately affected communities. Policies and practices can contribute to unequal access to care and disproportionate exposure to health risks, and disparities in morbidity and mortality from health conditions, including COVID-19; this extends to uptake of prevention practices, therapeutic trials, and vaccine acceptance [1-3].

The CEAL project is an NIH-wide effort to provide trustworthy information through active community engagement and outreach to the people hardest-hit by the COVID-19 pandemic, including African Americans, Hispanics/Latinx, and American Indians/Alaska Natives, with the goal of building long-lasting partnerships as well as improving diversity and inclusion in the research response to COVID-19. CEAL project teams were established in 11 states with the shared goal of quickly launching outreach efforts to help reduce the impact of COVID-19 on the most vulnerable populations and to evaluate these efforts through community-engaged research.

The Texas CEAL Consortium formed in August 2020 in response to the NIH call for statewide engagement. As of August 22, 2020, a total of 577,537 cases and 11,370 deaths due to COVID-19 had been documented in Texas [4]. Five Texas counties were among the top 50 counties in the USA with the greatest number of COVID-19 cases: Bexar, Dallas, Harris, Hidalgo, and Tarrant. There was an urgent need to establish effective community-engaged strategies to enhance education, awareness, access, and inclusion of underserved communities in COVID-19 prevention and treatment efforts and reduce COVID-19 disease disparities. The five Texas counties were chosen because of their high number of COVID-19 cases, high concentrations of minority communities (as high as 93% in Hidalgo county), existing community infrastructure, ongoing efforts to combat COVID-19, and the disproportionate burden of COVID-19. Three of the seven projects are located in Harris County, which has nearly 5 million residents, making it the most populous county in Texas and third most populous county in the USA. The overarching goal of the Texas CEAL Consortium is to understand factors that contribute to the disproportionate burden of COVID-19 in underserved communities and establish effective community-engaged strategies to enhance education, awareness, access, and inclusion of underserved communities in research designed to advance the prevention and treatment of COVID-19 and reduce disease disparities. The purpose of this paper is to describe the plans for the Consortium’s projects in response to COVID-19.

## Methods

The Texas CEAL Consortium organized seven projects that addressed Consortium research questions and focused on the five counties particularly affected. These projects were selected by the Consortium Executive Committee after several rounds of discussions involving academic and community partners and local/state agencies, based upon their potential for meaningful, rapid, and sustainable impact. The Consortium was administered through the University of North Texas Health Science Center (UNTHSC) and led by Jamboor K. Vishwanatha. The Consortium teams consist of academic partners, community partners, and local/county governmental agencies through Baylor College of Medicine, CAN DO Houston, Dallas Fort Worth (DFW) Community Health Worker (CHW) Association, Día de la Mujer Latina, Inc., MD Anderson Cancer Center, South Central Area Health Education Center (AHEC) San Antonio, Tarrant County Public Health, Texas A&M AgriLife Research, United Way of Tarrant County University of Houston, University of Texas Health San Antonio, University of Texas Rio Grande Valley (UTRGV), UNTHSC, and YMCA of Metropolitan Fort Worth.

The Consortium identified four objectives to help guide and focus the research and activities.

- **Objective 1**: Conduct community-engaged research to promote awareness and enhance knowledge to overcome COVID-19 misinformation in diverse racial and ethnic populations.
- **Objective 2**: Leverage existing community partnerships and proven local strategies to increase uptake and active participation of diverse racial and ethnic groups in evidenced-based COVID-19 prevention practices and therapeutic trials (e.g., ongoing and future vaccine trials).
- **Objective 3**: Disseminate science-based and targeted educational campaigns in diverse racial and ethnic communities and evaluate their impact on beliefs, attitudes, knowledge, and practices of COVID-19 prevention and treatment.
- **Objective 4**: Develop sustainable community-engaged organizational capacity by creating a repository of local community-centric, evidence-based COVID-19 knowledge products (i.e., best practices) targeted towards populations experiencing disproportionate burden of COVID-19.

The seven projects of the Texas CEAL Consortium will work toward the same overall goal and align with at least one of the objectives described above. Each Project Lead developed a team, selected populations of interest, and identified specific research objectives. To help organize the overall structure of the Texas CEAL Consortium, an Administrative Core will lead overall Consortium activities, be the liaison between NIH and Texas CEAL Consortium members, and compile metrics for all project activities. Additionally, a Data Management Coordinating and Infrastructure (DMCI) group will work closely with project investigators on study design, conduct, and analysis. Toward the aim of ensuring generalizability of findings, the DMCI group will work with both the Texas and national CEAL consortia to identify common data elements to ensure generalizability of measurements across consortia. All projects were submitted to and received Institutional Review Board approval. Project details are summarized in Table 1 and detailed below.

**Table 1.** Texas CEAL Consortium Projects

Project	Name	Lead	County	Consortium Research Objective	Population of Focus	Institutional Review Board
1	*Deliberative and Participatory Approaches to Addressing COVID-19 in African American and Hispanic Communities in Texas*	Bettina Beech, DrPH, MPH	Harris	1	African American, Hispanic/Latinx	University of Houston IRB (STUDY00002776)
2	*Unidos Contra COVID/United Against COVID*	Robert L. Ferrer, MD	Bexar	1, 3, 4	African American, Hispanic/Latinx, Asian	University of Texas Health Science Center at San Antonio IRB (C20200808E)
3	*Promoting Knowledge to Action in COVID-19 Prevention and Engagement among Underserved Communities*	Emily Spence, PhD, MSW	Tarrant	1, 2, 3	African American, Hispanic/Latinx	North Texas Regional IRB (IRB2020-134)
4	*Community Engagement Strategies for COVID-19 Prevention and Response in Underserved Communities in Hidalgo County*	Luis Torres-Hostos, PhD	Hidalgo	1, 3	Hispanic/Latinx	UTRGV IRB (IRB-20-0395)
5	*COVID Communications Resource Hub*	Rebecca A. Seguin-Fowler, PhD, RDN, LN, CSCS	Dallas	1, 2, 3, 4	African American, Hispanic/Latinx, LGBTQ, low literacy, older, rural, disabled	Texas A&M University IRB (IRB2021-0112)
6	*Engaging Blacks and Hispanics in COVID-19 Clinical Trials, Vaccine Uptake, and Educational Campaigns*	Lorna McNeill, PhD, MPH	Harris	1, 2, 3	African American, Hispanic/Latinx	University of Texas MD Anderson Cancer Center IRB (2021-0992)
7	*Identifying Health Inequity through Community and Personal Barriers to COVID-19 Interventions*	Jasmine J. Opusunju, DrPH, MSEd, MCHES, CPH	Harris	1, 2, 3, 4	African American, Hispanic/Latinx	Pearl IRB (21-CAND-101).

Abbreviations: CEAL: Community Engagement Alliance; COVID: coronavirus disease 19; IRB: Institutional Review Board; LGBTQ: lesbian, gay, bisexual, transgender, and queer; UTRGV: University of Texas Rio Grande Valley.

### Texas CEAL Consortium Projects

**Project 1**, “*Deliberative and Participatory Approaches to Addressing COVID-19 in African American and Hispanic Communities in Texas*,” is based in Harris County and led by Bettina Beech, DrPH, MPH. Project 1 focuses on African American and Hispanic/Latinx populations.

The Project 1 team will collaborate with the California CEAL team to conduct a two-site qualitative assessment of perspectives regarding “home-based” COVID-19 testing (home or near-home testing strategies depending on types of testing) and vaccine trial participation/vaccine acceptance from a diverse cohort of community residents in Houston, Texas through the use of deliberative methods with patient, provider, community, and population health leaders. Deliberative methods allow a participant-engaged approach that simultaneously gathers community input while providing education and then sequentially reassessing community participant views in the setting of new information that may counter rumors and misinformation [5]. The specific aims are to:

1) Use deliberative methods with African American and Hispanic/Latinx community opinion leaders to identify:

- Feasibility and acceptability of home-based testing including participant views on currently available technology (home-based collection with mailing of samples for analysis for results) and views regarding potential future point-of-care testing technology (home-based collection and analysis).
- Barriers (e.g., vaccine hesitancy) and facilitators (e.g., vaccine education) to uptake of COVID-19 vaccine trials and/or an approved vaccine and likely approaches to enhance community use of COVID-19 vaccine (e.g., vans, local community-based organizations, public health sites)**.**

2) Structure the content of the deliberative sessions around:

- Perception of risk and the relationship between risk perception and likely behavioral change.
- Identification of the content and treatment knowledge needed to enhance informed decision making about COVID-19 home-based testing and vaccine use/trial participation.
- Definition of successful implementation approaches of COVID-19 home-based testing and vaccine use from community perspectives.

**Project 2**, “*Unidos Contra COVID/United Against COVID*,” is based in Bexar County and led by Robert L. Ferrer, MD. Project 2 focuses on African American, Hispanic/Latinx, and Asian populations.

Leveraging community partnerships previously established in Bexar County, this project will apply a community-engaged strategy to increase affected communities’ access to timely, accurate guidance on reducing risk of COVID-19 infection. The initial phase will assemble community partners including the Bexar County Health Collaborative, the San Antonio Metropolitan Health District, grassroots groups, and places of worship. After establishing a shared understanding, through qualitative inquiry and survey responses, of the organizations’ and their communities’ initial conditions with respect to addressing COVID-19 risk, the Project 2 team will collaboratively develop tailored engagement and outreach strategies designed to reduce risk among their communities. Methods to accomplish this include technical assistance and budgeted dollars to enhance organizations’ capacity to develop and diffuse messaging salient to their communities. Additionally, a local social media firm will collaborate with community partners to develop, diffuse, and evaluate community-relevant messaging. Study outcomes to be evaluated include number of community organizations engaged, number of community members reached by each organization, implementation of the social media strategy, and social media engagement metrics. The team anticipates generalizable knowledge will emerge that is applicable to other communities disproportionately affected by COVID-19.

**Project 3**, “*Promoting Knowledge to Action in COVID-19 Prevention and Engagement among Underserved Communities*,” is based in Tarrant County and led by Emily Spence, PhD, MSW. Project 3 focuses on African American and Hispanic/Latinx populations.

The Project 3 team strategies will include weekly meetings of a Community-Based Participatory Research (CBPR) team to help establish iterative feedback cycles of data collection, outreach, and community education. Surveys and qualitative interviews will be used to 1) determine community-level misinformation and myths about COVID-19 and trust in sources of information and 2) assess knowledge, beliefs, attitudes, and fears about COVID-19 including vaccine hesitancy, vaccine acceptance, and willingness to participate in COVID-19 vaccine, therapeutic, and prevention trials in 12 primarily non-white and/or Hispanic/Latinx communities. The Project 3 team identified these communities based on the listening sessions and discussions with community partners and a data-driven analysis of zip codes where the highest prevalence of COVID-19 cases per 100,000 residents and death rates occurred. Thus, the target population is residents of Tarrant County living in neighborhoods with at least 75% African American or Hispanic/Latinx residents; income 45% below the median income of the county; and disparities associated with COVID-19 incidence, mortality, and testing site access.

Community partners for Project 3, DFW CHW Association, Tarrant County Public Health, United Way of Tarrant County, and YMCA of Metropolitan Fort Worth, will collaboratively facilitate all facets of community engagement, training, and educational campaigns. The CBPR team also includes community stakeholders, such as a barber and well-known grassroots activists. The CBPR team will have oversight over two rounds of surveys that include standardized CEAL items as well as items selected by the local team to measure COVID-19 impacts. Surveys will be supplemented with qualitative interviews of stakeholders to illuminate issues of COVID-19 mistrust and misinformation and identify potential engagement strategies. The educational campaigns will leverage existing community resources, local-centric unmet needs, existing guidance documents, and communication resources from the Centers for Disease Control and Prevention, NIH, and community partners. Given the dynamic nature of COVID-19 trends, iterative cycles of data collection and community engagement will allow the team to be responsive to changing needs in the region.

**Project 4**, “*Community Engagement Strategies for COVID-19 Prevention and Response in Underserved Communities in Hidalgo County*,” is based in Hidalgo County and led by Luis Torres-Hostos, PhD. Project 4 focuses on Hispanic/Latinx populations.

The UTRGV team from the Schools of Social Work and Medicine will partner with COVID Shield and the AHEC Program. COVID Shield is a volunteer-led effort that stems from the Rio Grande Valley COVID-19 Physician Task Force. Volunteers are deployed to engage with community members seeking COVID-19 testing in high-capacity testing sites (i.e., those testing 300 or more people per day). They disseminate up-to-date COVID-19-related prevention information, test information, and post-testing guidance. AHEC trains health professional students to reduce the significant shortage of primary healthcare providers in rural and medically underserved communities in the Rio Grande Valley and offers family medicine, acute and chronic illness care, health screenings, physical exams, immunizations, and preventive care. The three AHEC clinics serve over 2,100 patients. Through the CEAL project, COVID Shield Volunteers and AHEC COVID Scholars from social work, nursing, and other health professions at UTRGV will be trained to deliver a brief COVID-19 prevention intervention to individuals seeking COVID-19 testing at community testing sites and to AHEC patients. The Project 4 team will also share current COVID-19-related prevention information; information about COVID-19 testing and post-testing guidance; and vaccine information, including clinical trials information and encouragement to participate. The team will conduct baseline and follow-up assessments at 30, 60, 90, and 180 days. The initial intervention will be done at testing sites (COVID Shield), during phone contacts, or while patients wait for their medical appointments (AHEC). During follow-up surveys, AHEC COVID Scholars will also provide low-level case management to community members being contacted, including referrals for needed services (e.g., other health concerns, social services, and financial needs).

**Project 5**, “*COVID Communications Resource Hub*,” is based in Dallas County and led by Rebecca A. Seguin-Fowler, PhD, RDN, LN, CSCS. Project 5 focuses on the following populations: African American; Hispanic/Latinx; lesbian, gay, bisexual, transgender, and queer (LGBTQ); low literacy; older; rural; and people with disabilities.

The overall goals of the COVID-19 Communications Resource Hub are as follows:

1. In close collaboration with diverse community partners (The Concilio, Día de la Mujer Latina, Inc., Foremost Family Health Center, Los Barrios Unidos (a Federally Qualified Health Center [FQHC]), Martin Luther King, Jr. Family Clinic, and The Senior Source), conduct critical analysis and evaluation of COVID-19-related materials (e.g., educational content for community health educators; informational materials for consumers) and related adaptations and best practices, which will be used to populate a curated, web-based repository of COVID-19 products, with focused attention to serving groups differentially affected by COVID-19-related health disparities.
2. To conduct Clinical Trial Community Navigation (CTCN) training including a tailored COVID-19 module; the CTCN training includes culturally and linguistically proficient strategies and materials with respect to health literacy and social determinants of health.

To assess suitability of repository products for various groups experiencing disparities, the Project 5 team will convene a community advisory board, which will include members from existing partnerships. The team will also conduct document analysis and focus groups with community- and faith-based organizations. The repository website will provide a communications infrastructure as well as serve as the statewide visibility platform for the CEAL Consortium’s work – reaching all 254 Texas counties through the Texas A&M AgriLife network, enabling future expansion and rapid response to emerging issues of relevance to the community organization partner network and the communities they serve. In order to substantially increase recruitment and retention for minority participants in COVID-19 related trials, the team will partner with Día de la Mujer Latina, Inc. to conduct CTCN trainings, targeting recruitment within the counties of focus (Bexar, Dallas, Harris, Hidalgo, Tarrant), but offered statewide. Pre/post training evaluations will be used to assess participants’ knowledge gains and evaluate how misinformation and lack of transparency leads to mistrust.

**Project 6**, “*Engaging Blacks and Hispanics in COVID-19 Clinical Trials, Vaccine Uptake, and Educational Campaigns*” is based in Harris County and led by Lorna McNeill, PhD, MPH. Project 6 focuses on African American and Hispanic/Latinx populations.

The Project 6 team will work to design, evaluate, and disseminate educational messages designed to increase knowledge and improve attitudes and beliefs about COVID-19 clinical trials and vaccines. MD Anderson Cancer Center includes several community partners with whom the Project 6 team has longstanding relationships including several area African American and Hispanic/Latinx churches, schools, FQHCs, local CHW organizations (e.g., Pro Salud), neighborhoods (e.g., Acres Homes in Houston; 94% African American and Hispanic/Latinx), and cities (e.g., Cities of Baytown and Pasadena, both 60% racial/ethnic minorities). MD Anderson will create a series of educational messages and distribute them through various channels including video, print materials, social media, public service announcements, articles in African American newspapers, and other channels of information for African Americans and Hispanics/Latinx. Using previously collected COVID-19 data on communication channels and trusted sources, the team will work closely with existing partners, networks, and community members to develop new COVID-19 messages that specifically cover the topics of trials and vaccines. Additional formative data will be collected if needed to further contextualize messages. The resulting product will be a COVID-19 message toolbox that can be used by all community partners in the Greater Houston area and shared with partners across the state. Once developed and in partnership with organizational partners, the team will evaluate the effectiveness of the COVID-19 messages in various settings including FQHCs, churches, neighborhoods, and cities. Partners will select messages and communication channels that resonate with their respective populations/communities and test their effectiveness. Outcomes are changes in knowledge, attitudes, and beliefs; intention to participation in clinical trials and vaccine uptake; and enrollment in clinical trials and vaccine uptake, once available. Finally, the team will support clinical trial enrollment in COVID-19 research and vaccine trials, particularly trials at MD Anderson Cancer Center, Baylor College of Medicine, and other area COVID-19 research sites. MD Anderson has a clinical trial navigation program that helps reduce patient barriers to clinical trial participation and provides education, social support, and service referrals. Clinical trial navigators will identify potential trials for patients (MD Anderson only) and community participants (all other trials), work to navigate them to COVID-19 trials where they are potentially eligible, and support enrollment. Upon enrollment, navigators will also work to increase retention among trials that have follow-up visits.

**Project 7**, “*Identifying Health Inequity through Community and Personal Barriers to COVID-19 Interventions*,” is based in Harris County and led by Jasmine J. Opusunju, DrPH, MSEd, MCHES, CPH. Project 7 focuses on African American and Hispanic/Latinx populations.

This project will build on established community-driven strategies and diverse partnerships within CAN DO Houston and the FARO Initiative to examine factors contributing to the impacts of COVID-19 in underserved communities. Analysis of physical and social determinants of health within vulnerable communities in Houston will be conducted to develop community profiles and contextualize differences between communities that may contribute to awareness and attitudes toward COVID-19 vulnerability, prevention, and treatment. Existing relationships within these communities and partnerships will be leveraged to facilitate resident engagement through CHWs to assess attitudes, knowledge, perceptions, and awareness related to COVID-19. Based on responses about the impact of the COVID-19 pandemic, CHWs will facilitate access to essential wraparound resources selected by residents. Project 7 objectives are to:

1. Survey vulnerable communities to assess awareness, attitudes, fears, beliefs, and knowledge about risks of COVID-19 exposure, testing for the SARS-Cov-2 virus, and treatments and/or vaccines for COVID-19. The survey will be disseminated by CHWs via telephone.
2. Perform quantitative spatial analytics to examine the relationship between community contexts (social determinants of health), physical community metrics, and individual attitudes and awareness to COVID-19 in vulnerable communities.
3. Examine the relationship between community contexts (social determinants of health), physical community metrics, and individual attitudes and awareness to COVID-19. Through this analysis, we aim to understand the complex factors behind “access” and “acceptability” related to COVID-19 interventions and identify the barriers and opportunities to disrupt the spread of COVID-19 in vulnerable communities.

## Discussion

The overall mission of NIH CEAL projects is “to provide trustworthy, science-based information through active community engagement and outreach to the people hardest-hit by the COVID-19 pandemic, with the goal of building long-lasting partnerships as well as improving diversity and inclusion in our research response to COVID-19” [6]. Collectively, the Texas CEAL team projects serve as one alliance of interlinked community-engaged research projects across the United States to understand factors associated with engagement in COVID-19 research and to develop an evidence base for deployment of effective strategies to enhance awareness and uptake of preventive health measures to mitigate the pandemic and improve the outreach and inclusion in COVID-19 research among community-engaged partners in underserved communities. Community-engaged alliances are a key strategy for addressing health disparities, in terms of approaches to communicable diseases, such as COVID-19, as well as noncommunicable diseases. Texas CEAL projects will employ a variety of methods to serve the mission of improving engagement, outreach, and partnerships and increasing diversity and inclusion in the response to COVID-19, including conducting community-engaged research to enhance knowledge, leveraging existing community partnerships to increase participation in trials, disseminating educational campaigns, and developing sustainable community-engaged organizational capacity.

## Declarations

### Ethics Approval and Consent to Participate

All projects were submitted to and approved by Institutional Review Boards.

**Project 1:** University of Houston Institutional Review Board (STUDY00002776).

**Project 2:** University of Texas Health Science Center-San Antonio Institutional Review Board (HSC20200808E).

**Project 3:** North Texas Regional Institutional Review Board (IRB2020-134).

**Project 4:** University of Texas Rio Grande Valley Institutional Review Board (IRB-20-0395).

**Project 5:** Texas A&M University Institutional Review Board (IRB2021-0112).

**Project 6:** University of Texas MD Anderson Cancer Center Institutional Review Board (2021-0992).

**Project 7:** Pearl Institutional Review Board (21-CAND-201)

All participants provided written informed consent.

## Data Availability

Data sharing is not applicable to this article as no data sets were generated or analyzed during the current study.

## Consortium Members (in addition to the authors listed)

David Abraham, Rice University; Subhash Chauhan, University of Texas Rio Grande Valley; Robert Clark, University of Texas Health Science Center at San Antonio; Haley Delgado, CAN DO Houston; Enedina Enriquez, University of Texas Rio Grande Valley; Leyla Feize, University of Texas Rio Grande Valley; Venus Ginés, Día de la Mujer Latina, Inc.; Denise Hernandez, Dallas Fort Worth Community Health Workers Association; Lovell Jones, GROW Unity Resources for Living (FARO Initiative); Leah King, United Way of Tarrant County; J’Vonnah Maryman, Tarrant County Public Health; Kenny Mosely, YMCA of Metropolitan Fort Worth; Amy Ramirez, Operation COVID Shield Hidalgo County; Candace Robledo, University of Texas Rio Grande Valley; Usha Sambamoorthi, University of Texas Health Science Center; Erica Spears, University of Texas Health Science Center; Honora Swain-Ogbonna, CAN DO Houston; Melissa A. Valerio-Shewmaker, University of Texas Health Science Center Houston and University of Texas School of Public Health in Brownsville; Teresa Wagner, University of Texas Health Science Center; Paula Winkler, South Central Area Health Education Center San Antonio.
