# The Texas Community-Engagement Research Alliance Against COVID-19 in Disproportionately Affected Communities (TX CEAL) Consortium – CORRIGENDUM

**DOI:** 10.1017/cts.2025.88

**Published:** 2025-05-19

**Authors:** R. Seguin-Fowler, C. Amos, B. Beech, R. Ferrer, L. McNeill, J. Opusunju, E. Spence, E. Thompson, L. Torres-Hostos, J. Vishwanatha

This notice is to highlight that one of the funding grants was incorrectly published as: ‘**This research was, in part, funded by the National Institutes of Health (NIH) Agreement 1OT2HL156812-01 as part of the NIH Community Engagement Alliance (CEAL). The funding agency had no role in the design of the study or writing of the manuscript.**’

This funding grant should have been published as: ‘**This research was, in part, funded by the National Institutes of Health (NIH) Agreement 1OT2HL156812-01 as part of the NIH Community Engagement Alliance (CEAL) (OT2HL158287). The funding agency had no role in the design of the study or writing of the manuscript.**’

The authors apologise for this error.
